# Association between baseline pulse pressure and prognosis in critically ill patients: a retrospective cohort study

**DOI:** 10.1038/s41598-026-56390-z

**Published:** 2026-06-11

**Authors:** Yifan Gao, Wei Su, Yinshan Wu, Ying Chen, Senbin Hu, Zhendong Fang, Weipeng Huang, Jiali Mao, Kedi Gao, Minmin Chen, Qixun Luo, Shu Cao, Feng Guo

**Affiliations:** Department of Critical Care Medicine, Sir Run Run Shaw Hospital, Zhejiang University School of Medicine, Hangzhou, 310016 China

**Keywords:** Pulse pressure, Mortality, Retrospective, MIMIC, Biomarkers, Diseases, Health care, Medical research, Risk factors

## Abstract

To investigate the association between baseline pulse pressure (PP) and mortality in critically ill patients. This cohort study analyzed 22,035 ICU patients from the MIMIC-IV database. Baseline PP was defined as the first recorded value after admission. Outcomes included in-hospital, 180-day, and 365-day all-cause mortality. Feature importance was assessed using XGBoost. The relationship between PP and mortality was modeled with smooth curve fitting and threshold analysis. Multivariable logistic regression and survival analyses were performed to compare mortality risk across PP groups. Subgroup analyses examined effect modifications. The restricted mean survival time (RMST) analysis validated the robustness of the results. PP holds certain clinical significance in predicting ICU outcomes. A U-shaped relationship was identified, with an inflection point at 49 mmHg (95% CI 46–51). Patients were categorized into three groups: < 46, 46–51, and ≥ 51 mmHg. Using the 46–51 mmHg group as reference, both lower and higher PP groups showed significantly increased risks of in-hospital (*P* < 0.05) and long-term mortality (*P* < 0.05). Significant interactions were observed in the elderly and septic subgroups. RMST analysis further confirmed the stability of the results. Baseline PP nonlinearly predicts mortality risk in ICU patients, with the optimal range being 46–51 mmHg. This association is stronger in elderly and septic patients.

**Supplementary Information:**

The online version contains supplementary material available at 10.1038/s41598-026-56390-z.

## Introduction

The in-hospital mortality rate for critically ill patients approaches 30%. This not only imposes tremendous physical and psychological stress on patients but also places a substantial economic burden upon the healthcare system^1^. Within the current healthcare landscape, the growing demand for intensive care has led to strained hospital resources, thereby compromising patient treatment outcomes^2^. Although current therapeutic interventions include modalities such as mechanical ventilation and extracorporeal life support, these approaches demonstrate variable effectiveness across patient populations and are associated with high costs along with potential complications^3^. Therefore, a comprehensive investigation into risk factors associated with patient mortality is critically imperative.

In recent years, pulse pressure(PP) has garnered increasing attention among researchers^4^. PP—calculated as systolic blood pressure minus diastolic blood pressure—represents a value reflecting the complex hemodynamic interplay among stroke volume, heart rate, aortic compliance, and peripheral vascular tone^5^. The normal range of PP is generally considered to be between 40 and 60 mmHg^6,7^. Darne et al. first proposed PP as a risk factor for cardiovascular disease in their seminal publication^8^. Previous studies indicate that elevated PP may signal reduced arterial elasticity, whereas diminished PP is typically associated with impaired cardiac function and decreased stroke volume^9,10^. Existing studies have shown that PP is a readily available hemodynamic parameter associated with multiple adverse cardiovascular outcomes and provides better prognostic predictive value than mean arterial pressure^11^.

Beyond its association with cardiovascular diseases, PP is also linked to the prognosis of various diseases. For example, PP is used to predict the 1-year survival rate in kidney transplant recipients^12^. The relationship between baseline PP and hospital mortality in patients with non-traumatic subarachnoid hemorrhage is nonlinear, with an inflection point at 60 mmHg, whereas the nadir of risk occurs at baseline PP levels between 57 and 68 mmHg^13^. Among surgical patients, excessively low PP is associated with elevated postoperative complications^14^.

Changes in PP may reflect the status of the circulatory system, which is particularly important in critically ill patients. Thus, investigating the relationship between baseline PP and patient mortality in Intensive Care Unit (ICU) populations helps identify high-risk individuals and guides clinical management. However, existing literature on the correlation between PP and clinical outcomes remains limited, especially regarding its application in critically ill patients, warranting further investigation.

## Materials and methods

### Data source and study population

Medical Information Mart for Intensive Care IV database (MIMIC-IV v3.0), a standardized and publicly accessible ICU database, contains 94,458 ICU admission records from 2008 to 2022 at Beth Israel Deaconess Medical Center in Boston. The authors obtained authorization to access the database (ID: 42,775,134). All data were de-identified to protect patient privacy, with a waiver of the informed consent requirement^15^. The study adheres to the Strengthening the Reporting of Observational Studies in Epidemiology (STROBE) guidelines.

The data included demographic information, vital signs, laboratory tests and scoring systems, interventions, and clinical outcomes. Patients meeting the following criteria were included: (1) admitted to the ICU; (2) age ≥ 18 years. Exclusion criteria were as follows: (1) patients with multiple ICU admissions; (2) those lacking invasive blood pressure monitoring; (3) ICU stays lasting less than 1 day; (4) Outliers were excluded. Ultimately, 22,035 patients were enrolled with complete baseline data collected.

### Data extraction and data preprocessing

All relevant variables were extracted from medical records in the MIMIC-IV database using Structured Query Language (SQL) via PostgreSQL. We collected patient characteristics: age, gender, systolic blood pressure (SBP), diastolic blood pressure (DBP), heart rate, respiratory rate (RR), and peripheral oxygen saturation (SpO2). We collected severity scores: Acute Physiology Score III (APSIII), Age-adjusted Charlson comorbidity index (aCCI), Logistic organ dysfunction system (LODS), Model for End-Stage Liver Disease (MELD), Oxford acute severity of illness score (OASIS), and Sequential organ failure assessment (SOFA). We collected data on the use of vasopressors prior to ICU admission and ICU admission day interventions: vasopressor administration and invasive mechanical ventilation. We collected outcome measures: in-hospital mortality, 180-day mortality, and 365-day mortality. For missing data, multiple imputation was performed using the Random Forest method implemented in the R mice package(Missing Values and Outliers in Supplementary Table S1).

### Baseline PP level measurement and definition of outcomes

Baseline arterial blood pressure was defined as the first measurement recorded upon ICU admission. These values were obtained invasively via an arterial catheter. PP was calculated for each patient as the first SBP minus the first DBP^16,17^. The primary endpoint was all-cause in-hospital mortality, with secondary endpoints including 180-day and 365-day mortality.

### Study aims, scientific questions, and hypotheses

This study aims to investigate the relationship between baseline PP and in-hospital mortality, identify potential threshold effects and independent risk factors, and establish an evidence base for clinical practice.

Scientific question: Is there a significant association between baseline PP and in-hospital mortality as well as long-term survival among critically ill patients admitted to the ICU?

Scientific hypothesis: Baseline PP may serve as an independent predictor of prognosis in critically ill patients, and its relationship with mortality may follow a U-shaped nonlinear pattern—where both excessively high and low PP levels are associated with significantly elevated risks of death, whereas risk remains relatively lower within a certain normal range.

### Statistical analysis procedures

**Fig. 1:**
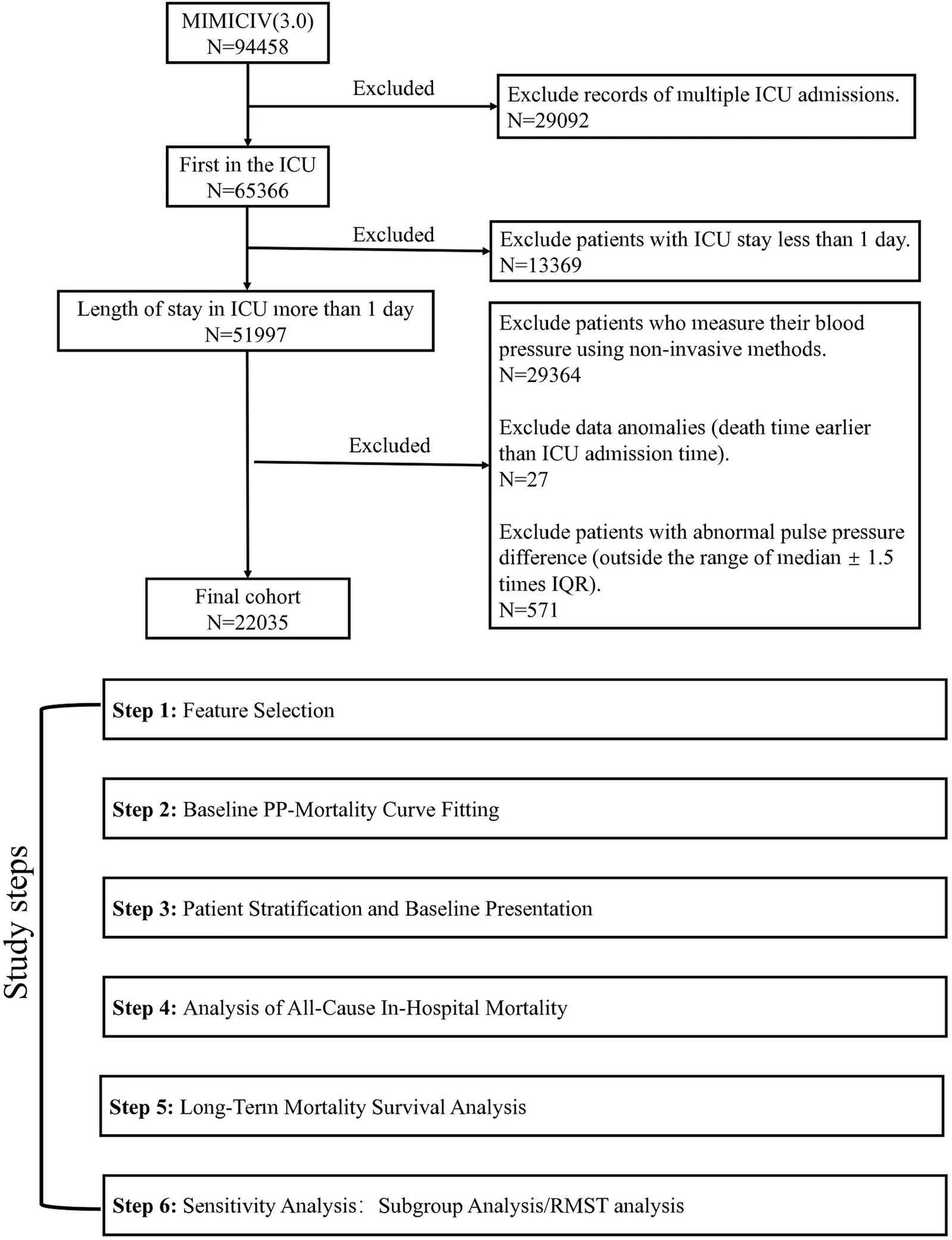
Study flowchart.

We proceeded with the analysis through the following six steps (Fig. 1):

**Step 1: Feature Selection.**

Before investigating the association between baseline PP and prognosis, we initially employed the eXtreme Gradient Boosting (XGBoost) machine learning algorithm for feature selection to determine its importance in prognostic modeling. In this regard, Shapley Additive explanations (SHAP) explanations were utilized for model interpretation (R, shapviz package, v0.10.2) to mitigate the inherent black-box nature of machine learning, thereby assisting clinicians in comprehending the results provided by the model. In this study, model development and validation were performed using a fivefold cross-validation framework. The XGBoost model was compared with the logistic regression model in terms of discrimination and calibration to evaluate its predictive performance for the outcome.

**Step 2: Baseline PP-Mortality Curve Fitting.**

Segmented univariable logistic regression and restricted cubic splines (RCS) analysis were performed to investigate threshold effects in the association between baseline PP levels and in-hospital mortality, with the bootstrap resampling method used to calculate the 95% CI of the inflection point. The optimal knots for the RCS were selected based on the minimum Akaike Information Criterion (AIC) value of the model.

**Step 3: Patient Stratification and Baseline Presentation.**

Based on the curve relationship identified in Step 2, patients were stratified according to the 95% CI of the PP inflection point, with baseline characteristics presented by group.

**Step 4: Analysis of All-Cause In-Hospital Mortality.**

Univariate and multivariate logistic regression analyses were performed to assess whether different PP groups serve as independent risk factors for in-hospital mortality in critically ill patients.

**Step 5: Long-Term Mortality Survival Analysis.**

Survival analysis was performed across PP groups for 180-day and 365-day mortality. Kaplan–Meier survival curves were generated and supplemented by both univariate and multivariate Cox proportional-hazards regression analyses.

**Step 6: Sensitivity Analysis.**

Subgroup analyses were conducted to evaluate the robustness of our findings, stratified by the following variables: age (< 65 years vs. ≥ 65 years), gender (male vs. female), use of vasoactive agents (yes vs. no), DBP (< 60 mmHg vs. ≥ 60 mmHg), and sepsis (yes vs. no).

In survival analysis, the validity of the hazard ratio (HR) relies on the proportional hazards assumption—i.e., that the HR remains constant over the study period—an assumption that often does not hold in practice. Therefore, in this study, we used the restricted mean survival time (RMST) as an alternative measure for re‑analysis. The RMST‑based difference does not depend on any model assumptions, remains valid throughout follow‑up, and provides a more stable estimate compared with median survival time^18^.

Continuous variables are expressed as mean ± standard deviation (SD) or median with interquartile range (IQR), while categorical variables are presented as numbers and percentages. Differences among the three PP groups were compared using one-way ANOVA for continuous variables and chi-square tests for categorical variables. Kaplan–Meier curves were analyzed using the log-rank test. A two-sided P-value < 0.05 was considered statistically significant for all analyses. All statistical analyses were performed using R version 4.5.1 (R Foundation for Statistical Computing, Vienna, Austria).

## Results

**Step 1: Feature Selection.**

We included 22,035 critically ill adult patients from the MIMIC-IV database with an initial ICU stay more than 1 day and complete invasive blood pressure records (Fig. 1). In comparing the XGBoost and logistic regression models for outcome prediction, XGBoost demonstrated superior performance in terms of both AUC and Brier score (Supplementary Figure S1).Using XGBoost, APSIII was identified as the strongest predictor of in-hospital mortality. SHAP-based feature importance analysis revealed that baseline PP contributed more to predicting mortality than body temperature, heart rate, SOFA, SIRS, and SpO2, though less than age and respiratory rate (Supplementary Figure S2). This suggests that PP holds certain clinical significance in predicting ICU outcomes.

**Step 2: Baseline PP-Mortality Curve Fitting.**

Segmented univariable logistic regression and RCS analysis established the relationship between baseline PP and in-hospital mortality(The number of knots for the RCS was set at 4 based on the minimum AIC value; for details, in Supplementary Table S2). Threshold analysis revealed a nonlinear association: Below 49-mmHg PP: Each 1-mmHg increase was associated with a 5% reduction in mortality risk (OR, 0.95; 95% CI 0.94–0.96; *P* < 0.001). At or above 49-mmHg PP: Each 1-mmHg increase corresponded to a 2% elevation in mortality risk (OR, 1.02; 95% CI 1.01–1.02; *P* < 0.001).

**Fig. 2:**
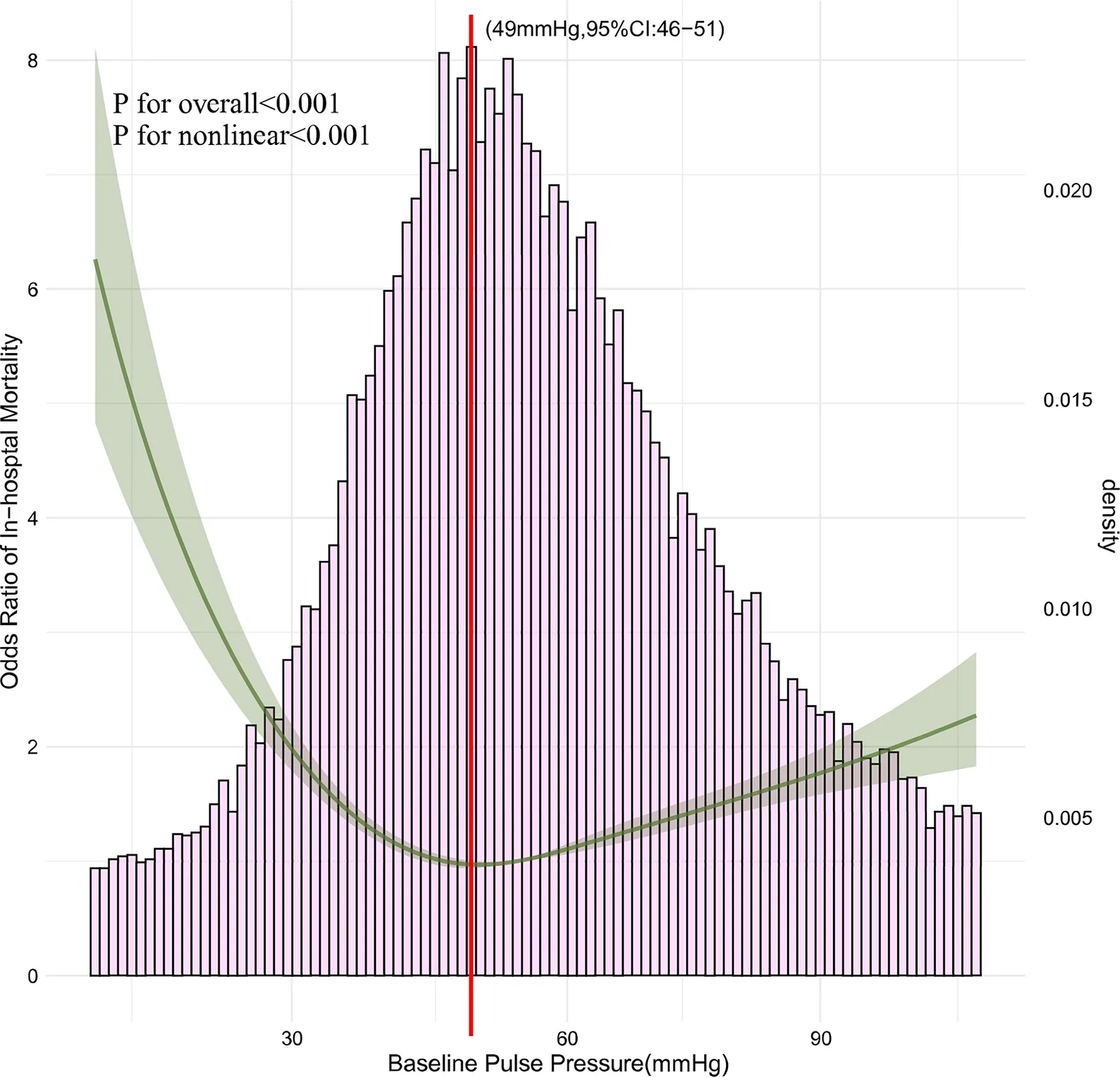
The relationship for the levels of baseline pulse pressure with in-hospital mortality.

Comparison of linear and segmented linear regression models by likelihood ratio test confirmed significant nonlinearity (*P* < 0.001), indicating a U-shaped relationship between baseline pulse pressure (PP) and mortality, which implies an increase in mortality with deviation from the cutoff value in either direction (Fig. 2 and Supplementary Table S3). Bootstrap-derived 95% CI for the inflection point was 46–51 mmHg.

**Step 3: Patient Stratification and Baseline Presentation.**

Table 1 describes the baseline characteristics of the included patients. The overall cohort had a mean age of 65.16 years, and 38.4% were female. Participants were stratified according to the 95% confidence interval of the inflection point for the relationship between baseline PP and in-hospital mortality (Group1: < 46 mmHg; Group2: 46 to 51 mmHg; Group3: ≥ 51 mmHg). Baseline table results indicated that patients with higher PP levels tended to be older and had higher aCCI. In contrast, those with lower PP levels exhibited higher APSIII, LODS, OASIS, and SOFA, along with a higher proportion of vasoactive drug use and invasive mechanical ventilation (all *P* < 0.001; Table 1).

**Table 1:** Population characteristics by quartiles of baseline pulse pressure.

PP_FIRST categorical	Overall	< 46	> = 46, < 51	> = 51	*P*-value
N	22,035	5030	2484	14,521	
Age, years	65.16 ± 14.97	63.50 ± 14.21	64.13 ± 13.98	65.91 ± 15.33	< 0.001
Gender					< 0.001
Female	38.4	35.4	33.5	40.3	
Male	61.6	64.6	66.5	59.7	
Heart rate, beats/min	85.14 ± 18.19	90.01 ± 19.13	85.65 ± 17.36	83.36 ± 17.68	< 0.001
RR, times/min	17.73 ± 5.57	18.19 ± 5.96	17.24 ± 5.35	17.65 ± 5.46	< 0.001
Temperature,℃	36.47 ± 0.89	36.39 ± 1.01	36.39 ± 0.84	36.51 ± 0.85	< 0.001
SpO2, %	97.92 ± 3.76	97.56 ± 4.54	98.23 ± 3.43	97.99 ± 3.51	< 0.001
APSIII	43.83 ± 22.64	48.08 ± 25.76	43.32 ± 22.73	42.44 ± 21.24	< 0.001
aCCI	4.51 ± 2.71	4.19 ± 2.59	4.14 ± 2.53	4.69 ± 2.76	< 0.001
LODS	4.83 ± 2.99	5.75 ± 3.18	4.94 ± 2.83	4.50 ± 2.88	< 0.001
MELD	13.38 ± 7.12	14.63 ± 7.60	13.47 ± 6.87	12.93 ± 6.93	< 0.001
OASIS	32.83 ± 8.9	34.42 ± 9.04	32.91 ± 8.37	32.26 ± 8.83	< 0.001
SOFA	5.40 ± 3.34	6.29 ± 3.52	5.75 ± 3.15	5.03 ± 3.24	< 0.001
SIRS	2.76 ± 0.90	2.93 ± 0.85	2.84 ± 0.85	2.68 ± 0.92	< 0.001
Vasopressors before ICU					< 0.001
NO	95.2	93.8	94.9	95.7	
YES	4.8	6.2	5.1	4.3	
Vasopressors					< 0.001
NO	48.6	33.1	35.7	55.2	
YES	51.4	66.9	64.3	43.8	
Invasive vent					< 0.001
NO	33.2	24.1	26.6	37.5	
YES	66.8	75.9	73.4	62.5	
Sepsis					< 0.001
NO	44.3	37.6	43.8	46.8	
YES	55.7	62.4	56.2	53.2	
Los hospital	11.82 ± 12.23	12.33 ± 13.28	11.21 ± 11.56	11.74 ± 11.95	0.002
Los ICU	4.96 ± 6.66	5.27 ± 7.11	4.22 ± 6.22	4.99 ± 6.56	< 0.001
In-hospital mortality					< 0.001
NO	89.7	87.2	92.6	90.1	
YES	10.3	12.8	7.4	9.9	
28-Day mortality					< 0.001
NO	88.9	86.6	91.7	89.2	
YES	11.1	13.4	8.3	10.8	
60-Day mortality					< 0.001
NO	86.7	84.7	89.8	86.9	
YES	13.3	15.3	10.2	13.1	
180-Day mortality					< 0.001
NO	83.3	81.5	87.4	83.2	
YES	16.7	18.5	12.6	16.8	
365-Day mortality					< 0.001
NO	80.6	79.3	84.8	80.4	
YES	19.4	20.7	15.2	19.6	

RR: respiratory rate; SpO2: Peripheral Oxygen Saturation; vasopressors before ICU: vasopressors use prior to ICU admission; Vasopressors: vasopressors use on the ICU admission day.

**Step 4: Analysis of All-Cause In-Hospital Mortality.**

Univariable analysis identified significant associations of in-hospital mortality with: Demographics: Age, Gender; Vital Signs: heart rate, RR, temperature, SpO2; Interventions: Vasopressor use, Invasive ventilation; Scoring Systems: APS III, LODS, MELD, OASIS, SIRS, SOFA, aCCI (All *P* < 0.05; see Supplementary Table S4).

Multivariable logistic regression analysis was performed to examine the association between baseline PP levels and in-hospital mortality, as summarized in Table 2. Three models were constructed: The crude model was unadjusted for covariates. Model I was adjusted for age, sex, heart rate, RR, temperature, and SpO2. Model II was further adjusted for vasopressors use before ICU, vasopressor use on ICU admission day, invasive ventilation, APSIII, LODS, MELD, OASIS, SIRS, SOFA, and aCCI.

**Table 2:** Multivariable analysis of factors associated with in-hospital mortality.

Pulse pressure, mmHg	OR(95% CI) *P*-value		
	Non-adjusted	Adjust Model I	Adjust Model II
Group1: < 46	1.84 (1.55, 2.18) < 0.001	1.56 (1.31, 1.87) < 0.001	1.38 (1.14, 1.68) 0.001
Group2: > = 46, < 51	1.0	1.0	1.0
Group3: > = 51	1.37 (1.17, 1.61) < 0.001	1.36 (1.16, 1.61) < 0.001	1.50 (1.25, 1.80) < 0.001

Adjust model I adjust for: Age; Gender; Heart rate; RR; Temperature ; SpO2.

Adjust model II adjust for: Age; Gender; Heart rate; RR; Temperature ; SpO2; Vasopressors before ICU, Vasopressors; Invasive vent; APSIII; LODS; MELD; OASIS; SIRS; SOFA; aCCI; Sepsis.

In all three models, a significant association was observed between the three baseline PP groups and in-hospital mortality, with Group2 as the reference (all ORs with *P* < 0.05). In the Model II, compared to Group2, patients in Group1 exhibited a 38% increase in in-hospital mortality risk, while those in Group3 showed a 50% increase in risk. The odds ratios for the three PP groups are presented graphically in Supplementary Figure S3.

**Step 5: Long-Term Mortality Survival Analysis.**

**Fig. 3:**
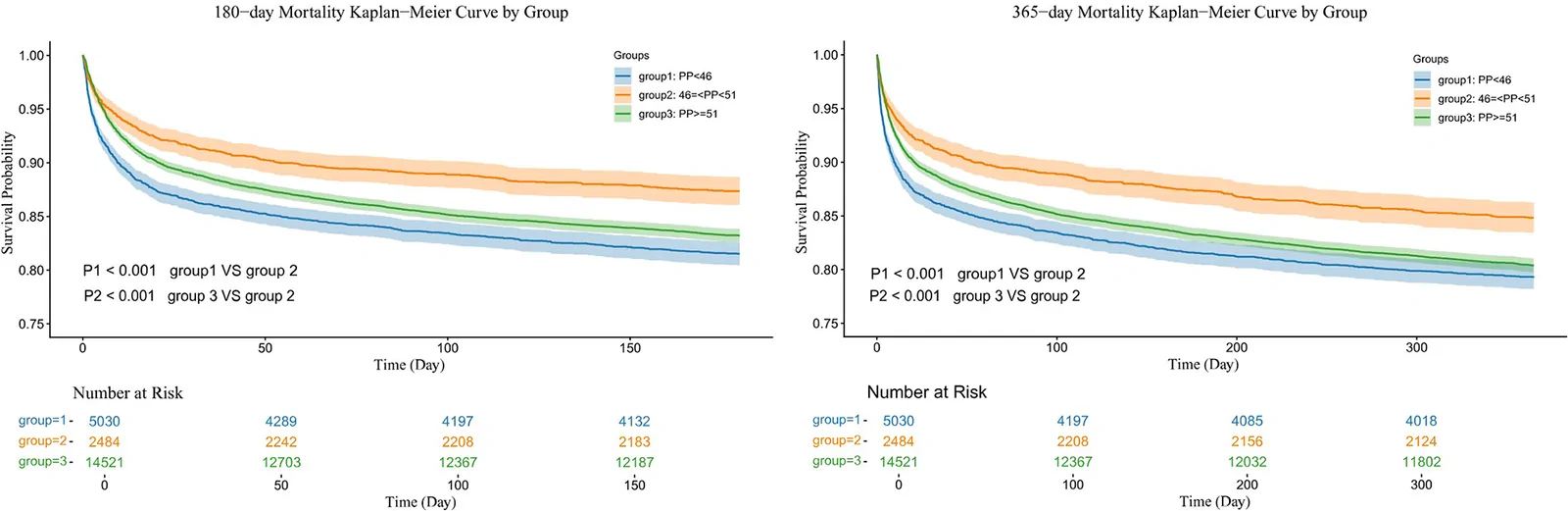
Kaplan–Meier analysis of Mortality Risk at 180 and 365 Days by Baseline Pulse Pressure groups.

Figure 3 presents Kaplan–Meier curves for the three groups, demonstrating superior survival in Group2 compared to Group1 and Group3 (*P* < 0.001).

We tested the proportionality assumption using Schoenfeld residuals; in some instances, the assumption was violated, we therefore interpreted the HR as weighted averages of the time-varying HR over the entire follow-up period^19^.

Results of the univariable Cox regression are detailed in Supplementary Table S5 and Supplementary Table S6. The adjusted multivariate HR results were as follows:

180-Day All-Cause Mortality: When stratified by baseline PP groups with Group2 as the reference: Group1: hazard ratio (HR), 1.17; 95% CI 1.03–1.33; *P* = 0.016; Group3: HR, 1.23; 95% CI 1.09–1.38; *P* = 0.001. Both showed significantly higher mortality risk (Supplementary Table S7).

365-Day All-Cause Mortality: When stratified by baseline PP groups with Group2 as the reference, both Group1 (HR, 1.13; 95% CI 1.00–1.27; *P* = 0.043) and Group3 (HR, 1.19; 95% CI 1.06–1.32; *P* = 0.002) showed significantly higher mortality risk at 365 days (Supplementary Table S7).

**Step 6: Sensitivity Analysis.**

We conducted stratified and interaction analyses to evaluate the associations between baseline PP levels (Group2 vs. Group1 and Group3) and long-term mortality risk (180-day and 365-day) across prespecified subgroups: gender (Male vs. Female), Age (≥ 65 vs. < 65 years), Vasoactive drug use (Yes vs. No), DBP (≥ 60 vs. < 60 mmHg), and Sepsis status (Non-sepsis vs. Sepsis).

In the subgroup analysis of 180-day and 365-day mortality risks, we observed an interaction effect between elderly and non-elderly patients, as well as between sepsis and non-sepsis patients. This indicates that the association identified in this study was more pronounced in elderly patients and septic patients (*P* < 0.05), thereby laying the groundwork for more targeted future research in these specific populations. Results are visualized in Supplementary Figure S4. To provide a more detailed characterization of the two subgroups, namely elderly patients and septic patients, we have added two separate tables summarizing their clinical characteristics, which are presented as Supplementary Table S8 and Supplementary Table S9 respectively.

Given that the proportional hazards assumption was not met for the groups in this study, we employed the RMST method to reassess the differences in mortality risk at 180 and 365 days among the three groups. According to the results in Figure S5, the estimated RMST for Group2 at the 180-day follow-up was 161.46 days. The RMST estimate for Group1 was 152.04 days. The RMST difference of 9.42 days (95% CI 6.78–12.06; *P* < 0.001) supported a longer survival time in Group2. The RMST estimate for Group3 was 155.72 days. Compared with Group2, the RMST difference was 5.74 days (95% CI 3.52–7.95; *P* < 0.001), also indicating longer survival in Group2. Similar results were observed at the 365-day follow-up (Supplementary Figure S6). These findings were statistically consistent with the results from the hazard ratio and log-rank tests, further supporting the robustness of the study.

## Discussion

This retrospective cohort study investigated the association between baseline PP and mortality in critically ill patients. The results revealed a U-shaped association between baseline PP and in-hospital mortality, indicating that both low and high PP levels were associated with an increased risk of death. This U-shaped pattern persisted for both 180-day and 365-day mortality, and sensitivity analysis using the RMST method suggested robust findings. Subgroup analysis further demonstrated that the U-shaped association was particularly pronounced in patients aged ≥ 65 years and in those with sepsis.

### Baseline PP as the exposure

The primary exposure variable in this study was the baseline PP, measured as the first PP recording following ICU admission. This selection was motivated by the need to capture the patient’s initial pathophysiological condition prior to confounding interventions commonly administered in the ICU—including fluid resuscitation and vasoactive drugs (e.g., vasopressors or inotropes)—which are known to influence hemodynamic parameters. Under conditions of low preload, such as early sepsis, fluid resuscitation augments ventricular end-diastolic volume and stroke volume (SV), leading to an increase in systolic pressure. Diastolic pressure may remain stable or decrease slightly owing to reduced systemic vascular resistance or enhanced microcirculatory perfusion, ultimately widening the PP^20^. In patients receiving vasoactive agents, reducing the dose of norepinephrine in cases of vasoplegia may lead to a decrease in PP^21^. An increase in norepinephrine dose was observed to significantly elevate PP^22^. In critically ill patients, the effects of therapeutic interventions on PP are complex, making it difficult to distinguish whether observed changes originate from the underlying disease or from external treatments. Therefore, selecting the baseline value allows for a more isolated assessment of the intrinsic association between PP and clinical outcomes. Furthermore, to minimize the influence of initial measurement error and enhance data integrity, we excluded recordings with extreme outliers or missing PP values. Additionally, we restricted inclusion to patients with complete hospital records available within 24 h of ICU admission, ensuring comprehensive clinical evaluation and treatment documentation.

In addition, recent studies have shown that evaluating the patient’s hemodynamics in combination with pulse pressure can more precisely regulate the use of pressor drugs^23^。Therefore, research on pulse pressure will have more substantial clinical significance.

### Association between PP and prognosis

Our study revealed a U-shaped association between baseline PP and both in-hospital and long-term mortality risks. Specifically, the range of 46 to < 51 mmHg (Group2) was associated with longer survival. PP is determined by SV, large artery compliance, systemic vascular resistance, and heart rate. Clinically, a low PP often indicates reduced SV, profound vasodilation, or hypovolemia, whereas a high PP typically reflects aortic stiffening or an elevated pulsatile load^7^. Therefore, the potential mechanism linking PP to mortality risk may be that a low PP is often associated with both low cardiac output and a loss of vascular tone. This combination can lead to hypoperfusion of vital organs (e.g., kidneys, brain, and heart), thereby increasing the risk of death^24^. Conversely, an elevated PP can promote the progression of atherosclerosis and increase the likelihood of plaque rupture and subsequent thrombosis^25^. Thus, the reduced survival observed in patients with elevated PP in our study population may be attributed to an increased incidence of cardiac, cerebral, and renal complications, particularly in relation to long-term mortality risk.

Consistent with findings in heterogeneous patient populations, Tokitsu et al. observed in HFpEF patients that the risks for cardiovascular and heart failure events were significantly elevated in the extreme low and high PP quintiles relative to the 45–74 mmHg reference group (*P* < 0.01)^26^. In patients with non-traumatic subarachnoid hemorrhage, PP exhibited a J-shaped association with in-hospital, ICU, and 28-day mortality, with an inflection point around 60 mmHg and an optimal range approximately 57–68 mmHg. Below 60 mmHg, each 5-mmHg increase in PP was associated with a decrease in mortality risk, whereas above 60 mmHg, each 5-mmHg rise was linked to an increased risk. Notably, the risk elevation was more pronounced at very high PP levels (≥ 105 mmHg)^13^. In patients with prehospital septic shock, an initial PP below 40 mmHg serves as a high-risk marker. An increase in PP during transport suggests effective resuscitation and is associated with a reduction in mortality risk.^24^

Multiple long-term follow-up studies have confirmed a robust association between baseline pulse pressure and long-term mortality. In the elderly population, pulse pressure has been demonstrated to be the best single blood pressure indicator for predicting mortality, with its predictive power slightly stronger than that of systolic blood pressure^27^. Studies on patients undergoing coronary artery bypass grafting (CABG) have found that an increased preoperative baseline pulse pressure is an independent predictor of reduced long-term survival postoperatively, whereas concurrently measured systolic blood pressure, diastolic blood pressure, or mean arterial pressure do not show such an association^25^. These evidences indicate that a single baseline pulse pressure value contains prognostic information beyond traditional blood pressure components.

### Prognostic value of PP in elderly and sepsis subgroups

Similar to our findings, a previous study demonstrated that elevated PP serves as an independent predictor of mortality in hospitalized older adults aged over 60 years^28^. Moreover, elevated PP serves as a biomarker of atherosclerosis. Atherosclerosis and sepsis share several pathophysiological features, including immune dysregulation, enhanced thrombogenesis, and systemic inflammation. Consequently, statin therapy has been associated with reduced risks of sepsis-related mortality through mitigating both atherosclerotic progression and inflammatory responses^29^.

A recent study defined a low PP phenotype as PP < 45 mmHg persisting for > 3 h after the first 72 h of ICU admission. Elderly patients with sepsis exhibiting this phenotype demonstrated a significantly elevated 28-day mortality risk^30^. From a physiological perspective, PP serves not only as an indicator of arterial vascular tone but also reflects dynamic changes in cardiac output^4,10^. In elderly patients, arterial stiffness increases with age, leading to elevated cardiac afterload and reduced cardiac output^31^. Therefore, a reduction in PP often suggests impaired cardiac function in elderly patients with sepsis.

### Limitation

Limitations of this study primarily stem from its retrospective design and the specific population selected. (1)Although the MIMIC-IV database provided a large cohort of ICU patients, which may introduce geographic bias and limit the generalizability of our findings. (2)Furthermore, despite the use of multiple statistical methods to adjust for potential confounders, unmeasured confounding factors cannot be fully eliminated. (3) Furthermore, as a retrospective study, inherent biases in data collection may affect the interpretation of the relationship between baseline pulse pressure and mortality. Additionally, the findings of this study merely indicate an association between pulse pressure and prognosis, without establishing causality, and further validation through randomized controlled trials is required. (4)While the findings of this study hold practical value in resource-limited settings and for rapid assessment, comprehensive patient evaluation in clinical practice still requires integration with illness severity scores (such as SOFA, APACHE, and Charlson) and continuous blood pressure monitoring. (5) Although this study incorporated information on vasopressor use prior to ICU admission, due to limitations in the database, data on whether fluid resuscitation was performed before ICU admission, as well as details of treatment received at other hospitals, are missing. Moreover, indications for ICU admission are also absent. These gaps might introduce certain biases into the results. (6) This study only included mortality as an outcome, and the association between pulse pressure and other cardiovascular complications still requires further research to supplement the key role of pulse pressure as a hemodynamic factor. Therefore, more detailed multicenter prospective studies are warranted to provide further evidence.

## Conclusion

We found a U-shaped relationship between baseline PP and in-hospital mortality in ICU patients, suggesting increased risk at both high and low PP extremes, particularly among the elderly and septic patients. The results highlight the clinical relevance of PP monitoring in critically ill populations, while also emphasizing the need to integrate additional severity scores for complete patient assessment.

## Supplementary Information

Below is the link to the electronic supplementary material.

Supplementary Material 1

## Data Availability

All data generated or analysed during this study are included in this published article.

## Abbreviations

aCCI: Age-adjusted Charlson comorbidity index
AIC: Akaike information criterion
APS III: Acute physiology score Iii
CI: Confidence interval
HR: Hazard ratio
ICU: Intensive care unit
IQR: Interquartile range
LRT: Likelihood ratio test
LODS: Logistic organ dysfunction system
MELD: Model for end-stage liver disease
MIMIC-IV: Medical information mart for intensive care IV database
OASIS: Oxford acute severity of illness score
PP: Pulse pressure
RCS: Restricted cubic splines
RMST: Restricted mean survival time
RR: Respiratory rate
SBP: Systolic blood pressure
SD: Standard deviation
SHAP: Shapley additive explanations
SIRS: Systemic inflammatory response syndrome
SOFA: Sequential organ failure assessment
SpO2: Peripheral oxygen saturation
SQL: Structured query language
STROBE: Strengthening the reporting of observational studies in epidemiology
SV: Stroke volume
XGBoost: Extreme gradient boosting
