# Supplementary material for: Association between baseline pulse pressure and prognosis in critically ill patients: a retrospective cohort study
Source: Sci Rep. 2026 Jun 11;16:26732. doi: 10.1038/s41598-026-56390-z (PMC13507337; doi:10.1038/s41598-026-56390-z)
Supplement: Supplementary file 1 [file 41598_2026_56390_MOESM1_ESM.docx]

**Table S1** Missing Values and Outliers

**TableS2** Values Across Various Knots of RCS

**Table S3** The relationship for the levels of baseline pulse pressure with in-hospital mortality

**TableS4** Univariable logistic regression analysis of factors associated with in-hospital mortality

**TableS5** Univariable Cox regression analysis of 180-Day mortality

**TableS6** Univariable Cox regression analysis of 365-Day mortality

**TableS7** Multivariable Cox regression analysis.

**Table S8** Population characteristics by quartiles of baseline pulse pressure, with a focus on the elderly subgroup.

**Table S9** Population characteristics by quartiles of baseline pulse pressure, with a focus on the septic subgroup.

**Figure S1** ROC curve comparison: XGBoost versus logistic regression for mortality prediction.

**Figure S2** Application of Machine Learning in Feature Selection.

**Figure S3** Multivariable analysis of factors associated with in-hospital mortality.

**Figure S4** Subgroup analyses for the association of baseline PP group with 180-day mortality and 365-day mortality.

**Figure S5** RMST with180-day mortality.

**Figure S6** RMST with365-day mortality.

| Variable Name | Lower Bound | Upper Bound | Number of Outliers | Number of Missing Values | Missing Proportion |
| --- | --- | --- | --- | --- | --- |
| Gender | - | - | - | 0 | 0% |
| Age | 27.681 | 105.09 | 500 | 0 | 0% |
| In-hospital mortality | - | - | - | 0 | 0% |
| Los hospital | -7.336 | 26.398 | 1867 | 0 | 0% |
| Los ICU | -4.315 | 11.085 | 2361 | 0 | 0% |
| Sepsis | - | - | - | 0 | 0% |
| Heart rate | 41.5 | 125.5 | 795 | 0 | 0% |
| RR | 5 | 29 | 974 | 16 | 0.07% |
| Temperature | 35.09 | 38.05 | 1871 | 1432 | 6.33% |
| SpO2 | 92.5 | 104.5 | 1539 | 6 | 0.03% |
| Pulse pressure | 11 | 107 | 571 | 0 | 0% |
| Vasopressors | - | - | - | 0 | 0% |
| Invasive vent | - | - | - | 0 | 0% |
| Vasopressors before ICU | - | - | - | 0 | 0% |
| APSIII | -11 | 93 | 891 | 0 | 0% |
| aCCI | -1.5 | 10.5 | 554 | 0 | 0% |
| LODS | -3 | 13 | 242 | 0 | 0% |
| MELD | -4 | 28 | 1192 | 0 | 0% |
| OASIS | 9 | 57 | 113 | 0 | 0% |
| SIRS | 0.5 | 4.5 | 186 | 0 | 0% |
| SOFA | -3 | 13 | 521 | 0 | 0% |
| 28-Day mortality | - | - | - | 0 | 0% |
| 60-Day mortality | - | - | - | 0 | 0% |
| 180-Day mortality | - | - | - | 0 | 0% |
| 365-Day mortality | - | - | - | 0 | 0% |

Table S1 Missing Values and Outliers

Lower Bound is defined as Q1 - 1.5 × IQR; Upper Bound is defined as Q3 + 1.5 × IQR.( IQR stands for Interquartile Range)

Table S2 AIC Values Across Various Knots of RCS

| nodes | AIC |
| --- | --- |
| 3 | 14897.49 |
| 4 | 14864.11 |
| 5 | 14866.52 |
| 6 | 14867.46 |
| 7 | 14868.31 |

Table S3 The relationship for the levels of baseline pulse pressure with In-Hospital mortality

| models | Per-1mmHg increase | |
| --- | --- | --- |
|  | OR (95%CI) | p value |
| Model I | 1.00 (1.00, 1.00) | 0.54 |
| One line effect |  |  |
| Model II |  |  |
| Turning point 95%CI (K, mmHg) | 49(46, 51) |  |
| pulse pressure < k | 0.95 (0.94, 0.96) | <0.001 |
| pulse pressure ≥ k | 1.02 (1.01, 1.02) | <0.001 |
| P value for LRT test |  | <0.001 |

LRT：Likelihood Ratio Test

TableS4 Univariable logistic regression analysis of factors associated with in-hospital mortality

|  | Statistics | OR(95%CI) *P-value* |
| --- | --- | --- |
| Group |  |  |
| Group1 | 5492 (24.92%) | 1.72 (1.46, 2.04) <0.0001 |
| Group2 | 2565 (11.64%) | 1.0 |
| Group3 | 13978 (63.44%) | 1.35 (1.16, 1.58) 0.0001 |
| Age, years | 65.16 ± 14.97 | 1.02 (1.02, 1.02) <0.001 |
| Gender |  |  |
| Female | 8463 (38.41%) | 1.0 |
| Male | 13572 (61.59%) | 0.80 (0.74, 0.88) <0.001 |
| Heart rate, beats/min | 85.14 ± 18.19 | 1.02 (1.02, 1.03) <0.001 |
| RR, times/min | 17.73 ± 5.57 | 1.10 (1.10, 1.11) <0.001 |
| Temperature,℃ | 36.47 ± 0.90 | 0.92 (0.88, 0.97) 0.001 |
| SPO2, % | 97.92 ± 3.76 | 0.92 (0.91, 0.93) <0.001 |
| APSIII | 43.83 ± 22.64 | 1.05 (1.04, 1.05) <0.001 |
| aCCI | 4.51 ± 2.71 | 1.21 (1.19, 1.23) <0.001 |
| LODS | 4.83 ± 2.99 | 1.40 (1.38, 1.42) <0.001 |
| MELD | 13.38 ± 7.12 | 1.12 (1.11, 1.13) <0.001 |
| OASIS | 32.83 ± 8.87 | 1.13 (1.13, 1.14) <0.001 |
| SOFA | 5.40 ± 3.34 | 1.31 (1.29, 1.33) <0.001 |
| SIRS | 2.76 ± 0.90 | 1.76 (1.66, 1.86) <0.001 |
| Sepsis |  |  |
| NO | 9771 (44.34%) | 1.0 |
| YES | 12264 (55.66%) | 4.44 (3.96, 4.97) <0.001 |
| Vasopressors before ICU |  |  |
| NO | 20975 (95.19%) | 1.0 |
| YES | 1060 (4.81%) | 3.34 (2.89, 3.86) <0.001 |
| Vasopressors |  |  |
| NO | 10705 (48.58%) | 1.0 |
| YES | 11330 (51.42%) | 1.82 (1.66, 1.99) <0.001 |
| Invasive vent |  |  |
| NO | 7320 (33.22%) | 1.0 |
| YES | 14715 (66.78%) | 3.30 (2.92, 3.72) <0.001 |
|  |  |  |

Group 1: <46 mmHg; Group 2: 46 to 51 mmHg; Group 3: ≥51 mmHg

TableS5 Univariable Cox regression analysis of 180-Day mortality

|  | | all | HR(95%CI, *P*-value) |
| --- | --- | --- | --- |
| Group | Group1 | 5030 (22.8%) | 1.53 (1.34-1.73, p<.001) |
|  | Group2 | 2484 (11.3%) |  |
|  | Group3 | 14521 (65.9%) | 1.35 (1.20-1.52, p<.001) |
| Age, years | Mean ± SD | 65.2 ± 15.0 | 1.03 (1.02-1.03, p<.001) |
| Gender | F | 8463 (38.4%) |  |
|  | M | 13572 (61.6%) | 0.83 (0.77-0.88, p<.001) |
| Heart rate, beats/min | Mean ± SD | 85.1 ± 18.2 | 1.02 (1.02-1.02, p<.001) |
| RR, times/min | Mean ± SD | 17.7 ± 5.6 | 1.07 (1.07-1.08, p<.001) |
| Temperature,℃ | Mean ± SD | 36.5 ± 0.9 | 0.97 (0.94-1.01, p=.135) |
| SPO2, % | Mean ± SD | 97.9 ± 3.8 | 0.95 (0.95-0.96, p<.001) |
| APSIII | Mean ± SD | 43.8 ± 22.6 | 1.03 (1.03-1.03, p<.001) |
| aCCI | Mean ± SD | 4.5 ± 2.7 | 1.25 (1.24-1.27, p<.001) |
| MELD | Mean ± SD | 13.4 ± 7.1 | 1.09 (1.09-1.09, p<.001) |
| OASIS | Mean ± SD | 32.8 ± 8.9 | 1.10 (1.09-1.10, p<.001) |
| SOFA | Mean ± SD | 5.4 ± 3.3 | 1.22 (1.21-1.23, p<.001) |
| SIRS | Mean ± SD | 2.8 ± 0.9 | 1.43 (1.38-1.49, p<.001) |
| Sepsis | No | 9771 (44.3%) |  |
|  | Yes | 12264 (55.7%) | 2.89 (2.68-3.12, p<.001) |
| Vasopressors before ICU | No | 20975 (95.2%) |  |
|  | Yes | 1060 (4.8%) | 2.80 (2.53-3.11, p<.001) |
| Vasopressors | No | 10705 (48.6%) |  |
|  | Yes | 11330 (51.4%) | 1.44 (1.35-1.54, p<.001) |
| Invasive vent | No | 7320 (33.2%) |  |
|  | Yes | 14715 (66.8%) | 1.77 (1.64-1.91, p<.001) |

Group 1: <46 mmHg; Group 2: 46 to 51 mmHg; Group 3: ≥51 mmHg

TableS6 Univariable Cox regression analysis of 365-Day mortality

|  | | all | HR(95%CI, *P*-value) |
| --- | --- | --- | --- |
| Group | Group1 | 5030 (22.8%) | 1.43 (1.27-1.61, p<.001) |
|  | Group2 | 2484 (11.3%) |  |
|  | Group3 | 14521 (65.9%) | 1.32 (1.19-1.47, p<.001) |
| Age, years | Mean ± SD | 65.2 ± 15.0 | 1.03 (1.03-1.03, p<.001) |
| Gender | F | 8463 (38.4%) |  |
|  | M | 13572 (61.6%) | 0.84 (0.79-0.89, p<.001) |
| Heart rate, beats/min | Mean ± SD | 85.1 ± 18.2 | 1.02 (1.02-1.02, p<.001) |
| RR, times/min | Mean ± SD | 17.7 ± 5.6 | 1.07 (1.06-1.07, p<.001) |
| Temperature,℃ | Mean ± SD | 36.5 ± 0.9 | 0.99 (0.95-1.02, p=.525) |
| SPO2, % | Mean ± SD | 97.9 ± 3.8 | 0.95 (0.95-0.96, p<.001) |
| APSIII | Mean ± SD | 43.8 ± 22.6 | 1.03 (1.03-1.03, p<.001) |
| aCCI | Mean ± SD | 4.5 ± 2.7 | 1.27 (1.25-1.28, p<.001) |
| MELD | Mean ± SD | 13.4 ± 7.1 | 1.09 (1.08-1.09, p<.001) |
| OASIS | Mean ± SD | 32.8 ± 8.9 | 1.09 (1.09-1.09, p<.001) |
| SOFA | Mean ± SD | 5.4 ± 3.3 | 1.20 (1.19-1.21, p<.001) |
| SIRS | Mean ± SD | 2.8 ± 0.9 | 1.36 (1.31-1.41, p<.001) |
| Sepsis | No | 9771 (44.3%) |  |
|  | Yes | 12264 (55.7%) | 2.55 (2.38-2.74, p<.001) |
| Vasopressors before ICU | No | 20975 (95.2%) |  |
|  | Yes | 1060 (4.8%) | 2.68 (2.43-2.96, p<.001) |
| Vasopressors | No | 10705 (48.6%) |  |
|  | Yes | 11330 (51.4%) | 1.35 (1.27-1.44, p<.001) |
| Invasive vent | No | 7320 (33.2%) |  |
|  | Yes | 14715 (66.8%) | 1.57 (1.47-1.68, p<.001) |

Group 1: <46 mmHg; Group 2: 46 to 51 mmHg; Group 3: ≥51 mmHg

Table S7 Multivariable Cox regression analysis.

|  | <46 | >=46, <51 | >=51 |
| --- | --- | --- | --- |
| 180-Day mortality | 1.17（1.03，1.33）*P*=0.016 | 1(Ref) | 1.23（1.09,1.38）*P*=0.001 |
| 365-Day mortality | 1.13（1.00，1.27）*P*=0.043 | 1(Ref) | 1.19（1.06,1.32）*P*=0.002 |

Adjust for: Age; Gender; Heart rate; RR; Sepsis; SPO2 Vasopressors before ICU; Vasopressors; Invasive vent; APSIII; MELD; OASIS; SIRS; SOFA; aCCI;

| Table S8 Population characteristics by quartiles of baseline pulse pressure, with a focus on the elderly subgroup. | | | | | |
| --- | --- | --- | --- | --- | --- |
| PP_FIRST categorical | Overall | <46 | >=46, <51 | >=51 | *P*-value |
| N | 12121 | 2490 | 1255 | 8376 |  |
| Age, years | 75.87 ±7.21 | 74.68 ±6.97 | 74.96 ±6.88 | 76.36 ±7.27 | <0.001 |
| Gender |  |  |  |  | <0.001 |
| Female | 39.7 | 36.3 | 34.7 | 41.5 |  |
| Male | 60.3 | 63.7 | 65.3 | 58.5 |  |
| Heart rate, beats/min | 83.23 ±17.29 | 88.04 ±18.38 | 84.31 ±16.05 | 81.63 ±16.86 | <0.001 |
| RR, times/min | 17.45 ±5.36 | 17.70 ±5.61 | 16.94 ±5.11 | 17.45 ±5.32 | <0.001 |
| Temperature,℃ | 36.37 ±0.87 | 36.29 ±0.93 | 36.28 ±0.81 | 36.40 ±0.85 | <0.001 |
| SpO2, % | 97.95 ±3.78 | 97.69 ±4.64 | 98.29 ±3.38 | 97.98 ±3.54 | <0.001 |
| APSIII | 45.29 ±21.73 | 48.59 ±24.65 | 44.98 ±22.28 | 44.36 ±20.60 | <0.001 |
| aCCI | 5.75 ±2.35 | 5.42 ±2.30 | 5.32 ±2.26 | 5.91 ±2.36 | <0.001 |
| LODS | 5.10 ±2.93 | 5.99 ±3.05 | 5.22 ±2.84 | 4.82 ±2.85 | <0.001 |
| MELD | 13.66 ±6.64 | 14.77 ±7.20 | 13.82 ±6.25 | 13.31 ±6.48 | <0.001 |
| OASIS | 34.29 ±8.64 | 35.62 ±8.86 | 34.18 ±8.27 | 33.90 ±8.59 | <0.001 |
| SOFA | 5.62 ±3.11 | 6.45 ±3.21 | 6.06 ±2.93 | 5.31 ±3.06 | <0.001 |
| SIRS | 2.70 ±0.91 | 2.87 ±0.86 | 2.79 ±0.86 | 2.63 ±0.92 | <0.001 |
| Vasopressors before ICU |  |  |  |  | 0.004 |
| NO | 94.9 | 93.7 | 94.7 | 95.3 |  |
| YES | 5.1 | 6.3 | 5.3 | 4.7 |  |
| Vasopressors |  |  |  |  | <0.001 |
| NO | 43.5 | 27.6 | 28.3 | 50.5 |  |
| YES | 56.5 | 72.4 | 71.7 | 49.5 |  |
| Invasive vent |  |  |  |  | <0.001 |
| NO | 32.5 | 22.9 | 26.9 | 36.2 |  |
| YES | 67.5 | 77.1 | 73.1 | 63.8 |  |
| Sepsis |  |  |  |  | <0.001 |
| NO | 44.4 | 38.3 | 43.3 | 46.4 |  |
| YES | 55.6 | 61.7 | 56.7 | 53.6 |  |
| Los hospital | 10.52 | 10.71 | 9.72 | 10.58 | 0.068 |
| Los ICU | 6.18 | 6.78 | 5.05 | 6.14 | <0.001 |
| In-hospital mortality |  |  |  |  | <0.001 |
| NO | 87.9 | 85.7 | 91.4 | 88.1 |  |
| YES | 12.1 | 14.3 | 8.6 | 11.9 |  |
| 28-Day mortality |  |  |  |  | <0.001 |
| NO | 86.7 | 85.0 | 90.4 | 86.7 |  |
| YES | 13.3 | 15.0 | 9.6 | 13.3 |  |
| 60-Day mortality |  |  |  |  | <0.001 |
| NO | 84.0 | 82.7 | 87.6 | 83.8 |  |
| YES | 16.0 | 17.3 | 12.4 | 16.2 |  |
| 180-Day mortality |  |  |  |  | <0.001 |
| NO | 79.6 | 78.9 | 84.8 | 79.1 |  |
| YES | 20.4 | 21.1 | 15.2 | 20.9 |  |
| 365-Day mortality |  |  |  |  | <0.001 |
| NO | 76.3 | 76.2 | 81.5 | 75.5 |  |
| YES | 23.7 | 23.8 | 18.5 | 24.5 |  |
| **RR:** respiratory rate; **SpO2:** Peripheral Oxygen Saturation; **vasopressors before ICU:** vasopressors use prior to ICU admission; **Vasopressors:** vasopressors use on the ICU admission day. | | | | | |

| Table S9 Population characteristics by quartiles of baseline pulse pressure, with a focus on the septic subgroup. | | | | | |
| --- | --- | --- | --- | --- | --- |
| PP_FIRST categorical | Overall | <46 | >=46, <51 | >=51 | *P*-value |
| N | 12264 | 3138 | 1397 | 7729 |  |
| Age, years | 65.23 ± 15.24 | 63.26 ± 14.74 | 64.14 ± 14.37 | 66.23 ± 15.50 | <0.001 |
| Gender |  |  |  |  | <0.001 |
| Female | 37.8 | 35.4 | 34.9 | 39.4 |  |
| Male | 62.2 | 64.6 | 65.1 | 60.6 |  |
| Heart rate, beats/min | 87.91 ± 19.56 | 93.04 ± 20.39 | 87.74 ± 18.46 | 85.86 ± 19.03 | <0.001 |
| RR, times/min | 18.37 ± 5.96 | 18.83 ± 6.40 | 17.73 ± 5.87 | 18.30 ± 5.77 | <0.001 |
| Temperature,℃ | 36.49 ± 1.00 | 36.43 ± 1.12 | 36.42 ± 0.94 | 36.53 ± 0.96 | <0.001 |
| SpO2, % | 97.65 ± 4.24 | 97.15 ± 4.97 | 98.01 ± 3.80 | 97.79 ± 3.96 | <0.001 |
| APSIII | 49.77 ± 24.63 | 53.89 ± 27.67 | 48.53 ± 25.09 | 48.32 ± 22.99 | <0.001 |
| aCCI | 4.65 ± 2.76 | 4.31 ± 2.69 | 4.26 ± 2.60 | 4.86 ± 2.79 | <0.001 |
| LODS | 5.77 ± 3.05 | 6.58 ± 3.24 | 5.69 ± 2.99 | 5.46 ± 2.93 | <0.001 |
| MELD | 15.03 ± 7.95 | 16.08 ± 8.35 | 14.88 ± 7.93 | 14.63 ± 7.75 | <0.001 |
| OASIS | 35.49 ± 8.46 | 36.59 ± 8.86 | 35.02 ± 8.25 | 35.13 ± 8.28 | <0.001 |
| SOFA | 6.46 ± 3.37 | 7.18 ± 3.62 | 6.56 ± 3.31 | 6.16 ± 3.23 | <0.001 |
| SIRS | 2.92 ± 0.87 | 3.06 ± 0.84 | 2.96 ± 0.83 | 2.85 ± 0.89 | <0.001 |
| Vasopressors before ICU |  |  |  |  | 0.001 |
| NO | 93.6 | 92.3 | 94.0 | 94.2 |  |
| YES | 6.4 | 7.7 | 6.0 | 5.8 |  |
| Vasopressors |  |  |  |  | <0.001 |
| NO | 37.2 | 24.7 | 27.4 | 44.1 |  |
| YES | 62.8 | 75.3 | 72.6 | 55.9 |  |
| Invasive vent |  |  |  |  | <0.001 |
| NO | 19.6 | 15.6 | 18.0 | 21.6 |  |
| YES | 80.4 | 84.4 | 82.0 | 78.4 |  |
| Los hospital | 14.19 ± 14.43 | 14.08 ± 14.77 | 12.92 ± 13.70 | 14.46 ± 14.40 | 0.001 |
| Los ICU | 6.74 ± 8.25 | 6.86 ± 8.44 | 5.60 ± 7.79 | 6.90 ± 8.23 | <0.001 |
| In-hospital mortality |  |  |  |  | <0.001 |
| NO | 84.6 | 82.1 | 88.6 | 85.0 |  |
| YES | 15.4 | 17.9 | 11.4 | 15.0 |  |
| 28-Day mortality |  |  |  |  | <0.001 |
| NO | 83.9 | 81.5 | 87.9 | 84.2 |  |
| YES | 16.1 | 18.5 | 12.1 | 15.8 |  |
| 60-Day mortality |  |  |  |  | <0.001 |
| NO | 81.0 | 79.0 | 85.0 | 81.0 |  |
| YES | 19.0 | 21.0 | 15.0 | 19.0 |  |
| 180-Day mortality |  |  |  |  | <0.001 |
| NO | 76.9 | 75.2 | 82.4 | 76.7 |  |
| YES | 23.1 | 24.8 | 17.6 | 23.3 |  |
| 365-Day mortality |  |  |  |  | <0.001 |
| NO | 74.1 | 72.8 | 79.8 | 73.6 |  |
| YES | 25.9 | 27.2 | 20.2 | 26.4 |  |
| **RR:** respiratory rate; **SpO2:** Peripheral Oxygen Saturation; **vasopressors before ICU:** vasopressors use prior to ICU admission; **Vasopressors:** vasopressors use on the ICU admission day. | | | | | |

Figure S1 ROC curve comparison: XGBoost versus logistic regression for mortality prediction.

The AUC of the two models were compared using DeLong’s test, with a *P* value < 0.05.

Figure S2 Application of Machine Learning in Feature Selection.

Figure S3 Multivariable analysis of factors associated with in-hospital mortality.

**Adjust I model adjust for:** Age; Gender; Heart rate; RR; Temperature ; SPO2

**Adjust II model adjust for:** Age; Gender; Heart rate; RR; Temperature ; SPO2, Vasopressors before ICU; Vasopressors; Invasive vent; APSIII; LODS; MELD; OASIS; SIRS; SOFA; aCCI; Sepsis.

Figure S4 Subgroup analyses for the association of baseline PP group with 180-day mortality and 365-day mortality.

**180-day mortality**

**365-day mortality**

Figure S5 RMST with180-day mortality.

Figure S6 RMST with365-day mortality.
